# Alterations of intestinal flora and the effects of probiotics in children with recurrent respiratory tract infection

**DOI:** 10.1007/s12519-019-00248-0

**Published:** 2019-04-24

**Authors:** Ke-Liang Li, Ben-Zhen Wang, Zi-Pu Li, Yi-Lei Li, Jing-Jing Liang

**Affiliations:** 1 Heart Center, Women’s and Children’s Hospital Affiliated to Qingdao University, Qingdao Women’s and Children’s Hospital, 6 Tongfu Road, Qingdao, 266034 Shandong China; 2 Department of Pediatrics, Rizhao People’s Hospital, 126 Taian Road, Rizhao, 276800 Shandong China; 3 Clinical Laboratory, Rizhao People’s Hospital, 126 Taian Road, Rizhao, 276800 Shandong China

**Keywords:** Children, Intestinal flora, Probiotics, Respiratory tract infections

## Abstract

**Background:**

Recurrent respiratory tract infection (RRTI) is a disease occurred frequently in preschool children.

**Methods:**

A total of 120 RRTI children were randomly divided into active group, remission group, intervention group and control group, meanwhile 30 healthy children were selected as the healthy group. Children in the intervention group were given oral *Bifidobaeterium* tetravaccine tablets (Live) for 2 months, while the control group received routine treatment. Stool sample were detected to analyze the bacterial strains. The occurrence of respiratory tract infection (RTI) was compared between different groups during 1 year follow-up.

**Results:**

Compared with the healthy group, the number of *Bifidobacteria* and *Lactobacilli* in the active group, remission group, intervention group and control group was significantly decreased (*P* < 0.05). The number of *Bifidobacteria* and *Lactobacilli* in the intervention group was significantly higher compared to other RRTI groups (*P* < 0.05). During the follow-up period, the average annual frequency of different acute RTI and use of antibiotics were significantly reduced (*P* < 0.05), the average duration of cough, fever and use of antibiotics at each episode were also significantly shortened (*P* < 0.05) in the intervention group compared to the control group.

**Conclusions:**

Children with RRTI are susceptible to intestinal flora imbalance. Oral probiotics can effectively improve the RRTI intestinal microecological balance in children and reduce the frequency of RTI.

## Introduction

Recurrent respiratory tract infection (RRTI) is one of the most common reasons for the pediatric visits and hospitalization. According to an epidemiological survey, the incidence rate of RRTI is about 20% in China, with an increasing tendency [1]. As previously reported, RRTI in children could increase the incidence of chronic respiratory disease in adulthood, and thereby cause permanent lung lesions [2]. Current findings have suggested that the different degrees of immunodeficiency or immature immune function, and specific or non-specific immune dysfunction in children with RRTI were major causes of RRTI [3]. Notably, the application of immunomodulatory agents is expected to improve the long-term prognosis of RRTI children.

Intestinal flora have a powerful regulatory effect on the human immune system. Human immune system could be regulated directly or indirectly by intestinal bacteria through increasing the number of extra-intestinal T cells, producing short-chain fatty acids, enhancing oral tolerance, or controlling inflammation [4]. Multiple studies have demonstrated that long-term use of probiotics could significantly reduce the risk of respiratory infections, and meanwhile could benefit for the fever, cough and administration duration of antibacterial agents in children [5–9]. Among these probiotics, *Lactobacillus* or *Bifidobacterium* is the most commonly used [10]. As known, the clinical effectiveness of various probiotics was different due to their regulatory effects on the human immune function [11]. *Bifidobaeterium* tetravaccine tablets (Live) is a probiotic preparation containing four kinds of strains (*Bifidobacterium infantis*, *Lactobacillus acidophilus, Enterococcus faecalis* and *Bacillus cereus*). Among them, the first three bacteria are the main intestinal probiotics. Besides, the *Bacillus cereus* can colonize in the intestine and consume oxygen to create an anaerobic environment for *Bifidobacteria*, thus promoting the growth and reproduction of anaerobic bacteria such as *Bifidobacteria*.

Based on the above reported studies, we aimed to investigate the clinical effects of *Bifidobaeterium* tetravaccine tablets (Live) on RRTI, and expected to provide a highly effective way for the prevention and treatment of RRTI.

## Methods

### Ethical statement

Ethical approval for the study has been obtained from the Ethics Committee of Rizhao People’s Hospital (code number: rzrmyy2015llpj002). In addition, written informed consent was obtained from parents or legal guardians of all children prior to any study-related procedure in the study.

#### RRTI patients

A total of 120 RRTI children admitted to the People’s Hospital of Rizhao between July 2015 and December 2015 were recruited into this study. Patients were eligible for enrollment in the RRTI group if they were 11 years of age or younger and had a clinical diagnosis of RRTI. The diagnosis of RRTI was performed according to the criteria issued by the Pediatric Society, Chinese Medical Association in 2007 [12]. Additionally, the children with RRTI were eligible for enrollment if they had no history of other diseases and no history of using antibiotics and probiotics. Exclusion criteria were as follows: (1) patients who were diagnosed with RRTI caused by organic or congenital lesions, or primary immunodeficiency; (2) patients with mental illness; (3) during the follow-up, patients who were not received *Bifidobaeterium* tetravaccine tablets (Live) in accordance with the prescribed method for a period greater than 20% of the follow-up duration or were given other immunosuppressive drugs or withdrew from the study.

All children with RRTI included in this study were equally assigned by a random number table into 4 groups: (1) remission group included 30 children without history of respiratory infection for more than 1 week; (2) Active group included 30 children who had fever, cough, stuffy nose, runny nose and other symptoms of RTI or those who had these symptoms disappear within 3 days; (3) Intervention group included 30 children with RRTI who had fever, cough, stuffy nose, runny nose and other symptoms of respiratory tract infection disappear for more than 4 days. They were given oral *Bifidobaeterium* tetravaccine tablets (Live) for 2 months after this episode; (4) Control group included 30 children who had fever, cough, stuffy nose, runny nose and other symptoms of respiratory tract infection. They were given conventional symptomatic treatment.

Stool specimens in the four groups were collected to analyze the intestinal flora at the corresponding time points. For the active group and the remission group, stool specimens were collected within 1–2 days after enrollment, while for the intervention group and the control group, were collected within 1 week after oral administration of *Bifidobaeterium* tetravaccine tablets (Live) or conventional symptomatic treatment, respectively.

#### Healthy control

Totally 30 children underwent a physical examination in the Department of Children Healthcare, People’s Hospital of Rizhao were enrolled as the healthy control (15 boys and 15 girls). Inclusion criteria were as follows: (1) children younger than 11 years old; (2) healthy subjects with no history of genetic disease, heart, liver, kidney and blood diseases; (3) subjects who had no history of upper RTI, acute and chronic gastrointestinal disease more than 1 week before specimens collection; (4) subjects who had no history of using antibiotics and probiotics, and had normal stool indicators by routine examination.

### Development of RRTI questionnaire

Questionnaires were distributed to the control and intervention groups. The questionnaire was compiled by a trained and full-time doctor (reviewed the patient’s previous medical records and treatment) and the guardians of children. The items mainly included: incidence, etiology, incentives, frequency of hospitalizations, frequency and timing of administration of antibiotics and shortest interval of acute upper RTI, acute bronchitis or pneumonia within 12 months from the time of survey, as well as the presence or absence of underlying diseases, etc.

### Stool specimens collection

Two samples (≈ 3 cm^3^) were collected into two collection tubes containing colony stabilizer, respectively. The child defecated on an opened aseptic paper-plastic bag (prepared by the People’s Hospital of Rizhao), then an appropriate amount of stool was transferred into the tube using a disposable sterile tongue depressor. Next, the tube was immediately placed in an ice bucket and stored at − 80 °C within 6 hours.

### Genome DNA extraction and quantitative PCR

Genome DNA was extracted from the stool samples using stool DNA rapid extraction Kit (Aidlab Biotechnologies Co., Ltd. Beijing, China) according to the manufacturer’s protocol. Primers were designed according to 16SrRNA sequences of *Bifidobacteria*, *Lactobacillus*, *Escherichia coli*, respectively; and then were verified in BLAST database. The upstream and downstream primer sequences were as follows: *Bifidobacteria* 5′-CTC CTG GAA ACG GGT GG-3′, 5′-CTC CTG GAA ACG GGT GG-3′ (550 bp); *Lactobacillus* 5′-CTG ATG TGA AAG CCC TCG-3′, 5′-GAG CCT CAG CGT CAG TTG-3′ (166 bp); *Escherichia coli* 5′-CTG ATG TGA AAG CCC TCG-3′, 5′-CGG GTA ACG TCA ATG AGC AAA-3′(95 bp). Primers were synthesized by Shanghai Sangon Biotech Co., Ltd.

The PCR reaction was amplified using a Stratagene M × 3000P PCR machine (Agilent Technologies, Inc.). The stool DNA samples were subjected to conventional PCR using each of the bacterial primers, and the amplified products were subjected to PAGE electrophoresis, followed by comparing with the standard molecular weight mark to verify them. The fluorescent quantified PCR was performed to obtain the three bacteria number of all the DNA samples. Consequently, the solubility curves of fluorescence quantitative PCR of the three bacteria were developed to verify the PCR amplification products. The standard strains and colonies of the tree bacteria were serially diluted (10^2^–10^10^ cfu/mL), followed by fluorescence quantitative PCR to detect the sensitivity of the fluorescent quantitative PCR.

### Treatment

*Bifidobaeterium* tetravaccine tablets (Live) was administered orally for 2 months. For children < 1 year, 2 tablets were given each time (twice daily), while for those ≥ 1 year, 3 tablets were used each time (twice daily). During follow-up, children with RTI were given conventional treatment, and based on this, anti-infective treatment were rationally selected according to the disease condition and the age of children. Meanwhile, no other immunomodulatory agents and probiotic preparations were allowed during the follow-up. Rational use of antimicrobial methods, such as strict application criteria, reasonable choice of the timing given (antibiotics and *Bifidobaeterium* tetravaccine tablets (Live) interval of more than 2 hours), and shorten the course of treatment, were given to both of the intervention group and control group. Besides, there was no difference in the choice, time, type and course of antibiotic application between the two groups.

### Clinical evaluation

According to the previous report, clinical efficacy was divided into four categories including [2]: (1) Cure: patients did not have recurrence of respiratory tract infection or occasional upper respiratory tract infection that was cured without any treatment within 1 year; (2) Marked effect: within 1 year, patients had < 3 times of episodes of respiratory tract infection, with effectively improved conditions that only required oral drugs; (3) Effectiveness: within 1 year, patients had < 5 times episodes of respiratory tract infection, with alleviated symptoms; (4) Ineffectiveness: within 1 year, the frequency of episodes of respiratory tract infection did not change or were increased.

### Statistical analysis

Statistical analyses were performed using SPSS19.0 software (IBM, New York, USA). Mean ± standard deviation (SD) was used to represent the continuous variables. Qualitative data were described by number or percentage. Quantitative PCR data were performed using logarithm process (1og copies/g) and were expressed as mean ± SD. For multiple sets of data, one-way analysis of variance was performed, multiple comparisons of strains among different groups were conducted using *t* test, and percentage data were compared using *χ*^2^ test. All statistical tests were two-sided tests, and *P* < 0.05 was considered statistically significant.

## Results

### Initial patient characteristics

The baseline characteristics of patients in the five groups were analyzed. The gender (male/female), age (month), body weight (kg) and body surface area (m^2^) of children in five groups were, respectively, as follows: 16/14, 43.17 ± 25.51, 15.6 ± 4.8, 0.65 ± 0.05 in the remission group; 17/13, 47.70 ± 29.38, 16.4 ± 5.4, 0.68 ± 0.10 in the active group; 14/16, 46.60 ± 30.20, 16.2 ± 5.5, 0.67 ± 0.05 in the intervention group; 14/16, 44.80 ± 24.09, 16.0 ± 4.5, 0.66 ± 0.28 in the control group; and 15/15, 50.80 ± 27.19, 17.0 ± 4.9, 0.69 ± 0.02 in the healthy group. No significant difference in patients’ characteristics (age, gender, body weight, and body surface area) was found among the five groups (*P* > 0.05).

### Validation, standard curves and solubility curves of PCR products-amplified fragments of *Bifidobacteria*, *Lactobacilli* and *Escherichia coli*

**Fig. 1:**
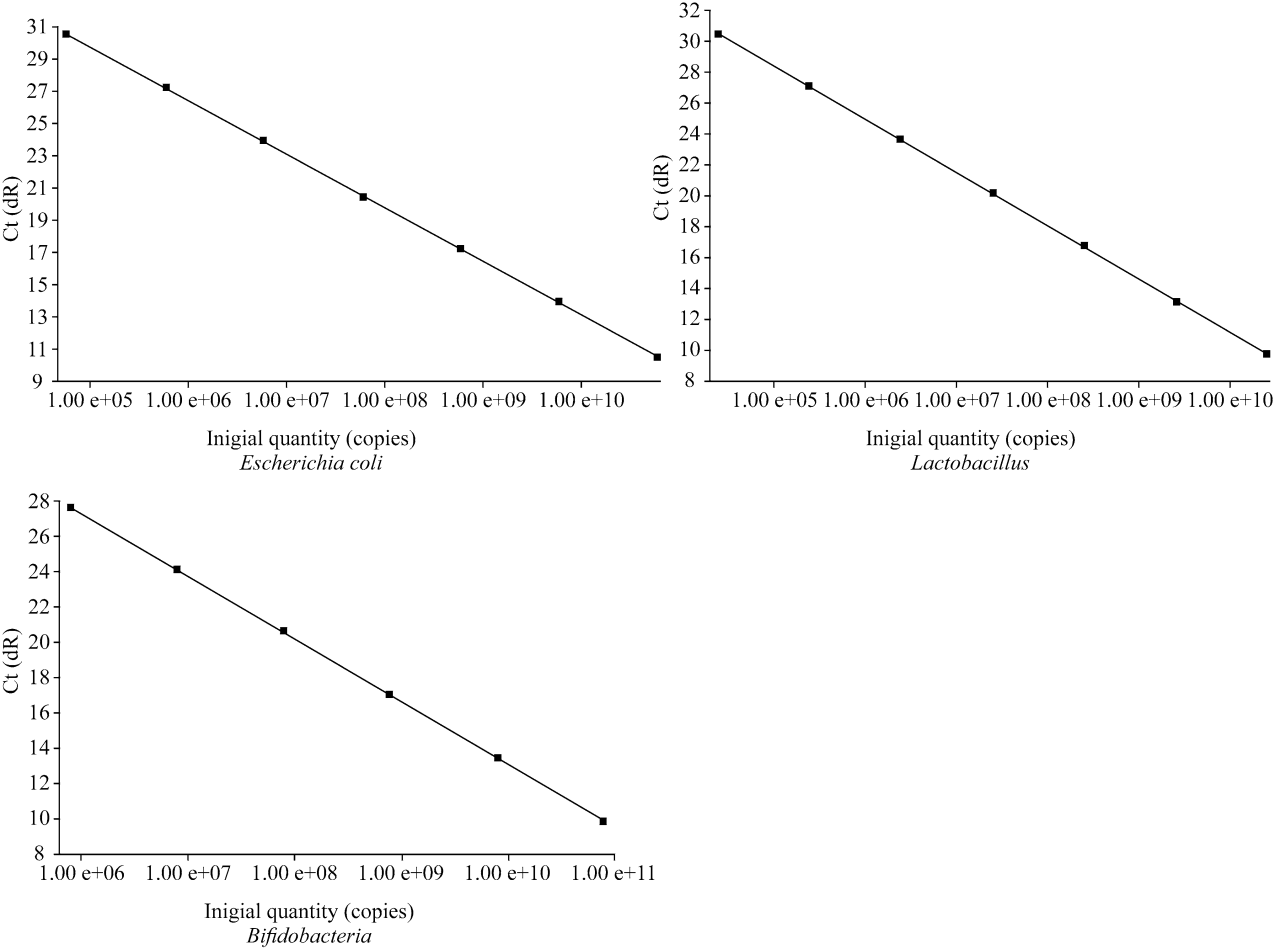
Standard curves of fluorescence quantitative PCR of *Bifidobacteria*, *Lactobacilli* and *Escherichia coli*

**Fig. 2:**
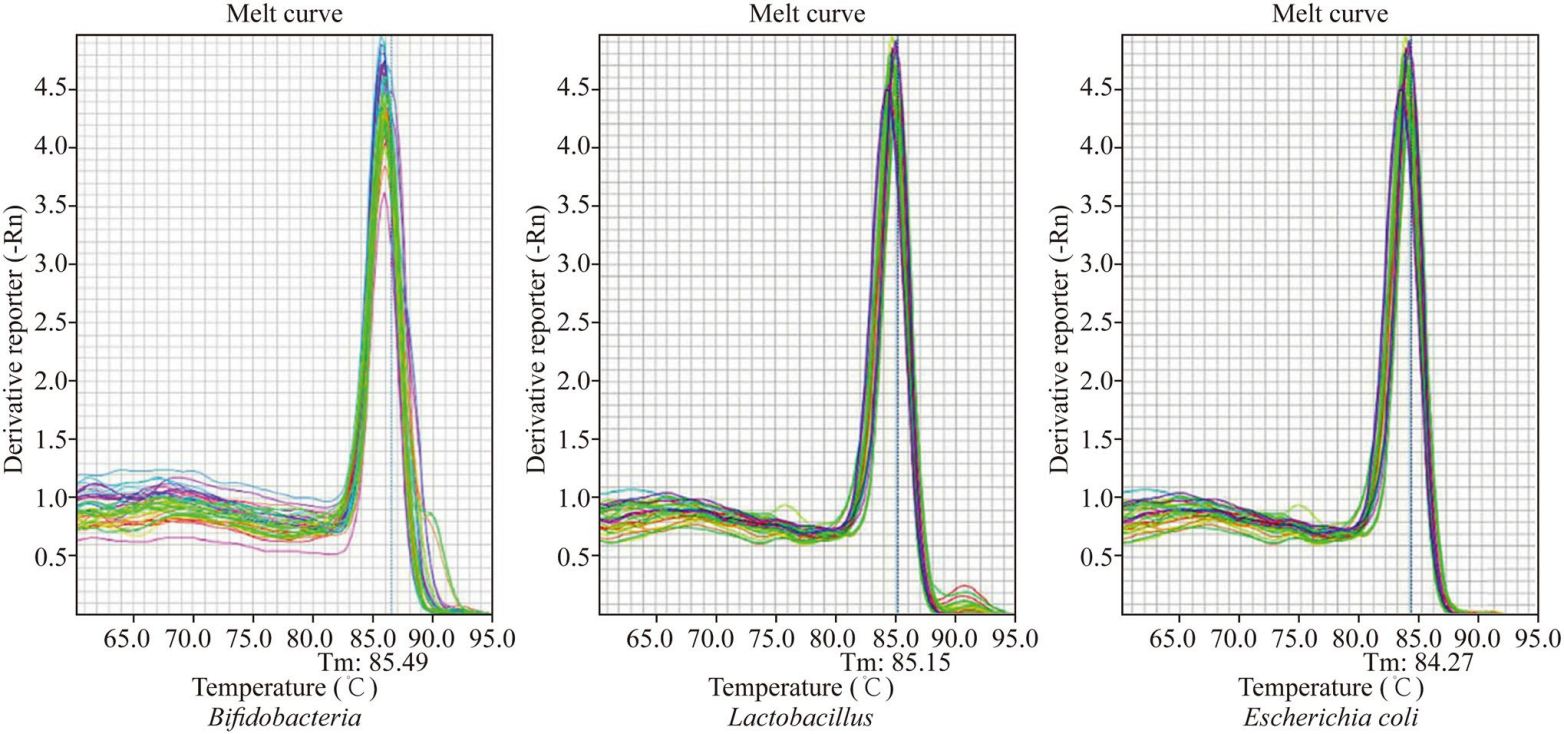
Solubility curves of fluorescence quantitative PCR of *Bifidobacteria*, *Lactobacilli* and *Escherichia coli*

PAGE electrophoresis of amplified fragments of *Bifidobacteria, Lactobacilli* and *Escherichia coli* revealed that the PCR amplification products consisted of a single band and had a length consistent with the expected length of the DNA fragment; these data confirmed that each primer had a good specificity. Fluorescence quantitative PCR of the serial dilutions of the three bacterial strains still showed characteristic growth curves at 100 cfu/mL, which indicated a good detection sensitivity (Fig. 1). The solubility curves from all three bacteria had a single peak, no nonspecific amplification, primer dimer and other interference phenomena were observed (Fig. 2).

### Comparison of fluorescence quantitative PCR results of intestinal flora among different groups

As shown in Table 1, the number of intestinal *Bifidobacteria* and *Lactobacilli* in the remission group, active group and control group was significantly decreased compared with the healthy group (*P* < 0.05). In addition, the number of intestinal *Bifidobacteria* and *Lactobacilli* in intervention group was significantly higher compared to control group, active group and remission group (*P* < 0.05), and was slightly higher compared to healthy group (*P* > 0.05). Meanwhile, the number of intestinal *Bifidobacteria* and *Lactobacilli* in the active group was slightly smaller compared to the remission group with statistically insignificant difference (*P* > 0.05), while the number of *Escherichia coli* was increased compared to the remission group, intervention group and healthy group (*P* < 0.05).

**Table 1:** Comparison of fluorescence quantitative PCR results of intestinal flora among different groups (mean ± SD, log copies/g)

Groups	Number of cases	*Bifidobacteria*	*Lactobacilli*	*Escherichia coli*
Healthy	30	8.89 ± 0.49	8.52 ± 1.42	7.42 ± 1.11
Remission	30	7.99 ± 1.16*	7.76 ± 0.45*	7.56 ± 1.30
Active	30	7.87 ± 1.39*	7.51 ± 0.55*	8.11 ± 1.24*^†^
Control	30	7.91 ± 1.23*	7.70 ± 0.54*	7.78 ± 1.39
Intervention	30	8.93 ± 1.08^†‡§^	8.71 ± 1.06^†‡§^	7.44 ± 1.22^‡^
*F* value		7.1191	10.9949	1.5841
*P* value		<0.0001	<0.0001	0.1816

Compared with the healthy group ^*^P < 0.05, compared with the remission group ^†^*P* < 0.05, compared with the active group ^‡^*P* < 0.05, compared with the control group ^§^*P* < 0.05

### Follow-up results of RRTI control and intervention groups

As shown in Table 2, the annual average of acute upper RTIs, acute (branch) bronchitis, pneumonia and the total number of antibiotics use per subject in the intervention group were significantly lower than those in the control group (*P* < 0.05). Besides, the average use of antibiotics, the duration of cough and the duration of fever per episode of RTI were significantly reduced compared to the control group (*P* < 0.05). Total of 16 subjects (22 times) in the control group required hospitalization, which was significant higher than that in the intervention group (4 subjects, 5 times) (*P* < 0.05). The total effectiveness rate of intervention group (*n* = 27, 90%) was significant higher than that of control group (*n* = 10, 33.3%).

**Table 2:** Follow-up results of respiratory tract infection in RRTI control and intervention groups

Items	Control group (*n* = 30)	Intervention group (*n* = 30)	*t* value	*P* value
Average number of acute upper respiratory tract infections (times/y)	4.03 ± 4.95	1.93 ± 2.12	2.1360	0.0185
Average number of acute bronchitis or bronchitis (times/y)	0.83 ± 0.71	0.37 ± 0.75	2.4396	0.0089
Average number of pneumonia (times/y)	1.47 ± 2.12	0.40 ± 1.41	2.3018	0.0125
Average number of use of antibiotics (times/y)	2.17 ± 3.54	0.63 ± 1.47	2.2006	0.0159
Duration of use of antibiotics for each onset (d)	6.12 ± 2.87	4.13 ± 2.54	2.8440	0.0031
Duration of fever for each onset (d)	3.57 ± 1.93	2.03 ± 1.45	3.4942	0.0005
Duration of cough for each onset (d)	6.53 ± 3.39	4.02 ± 1.85	3.5598	0.0004

### Adverse reactions

There were no cases of adverse events and drug-related adverse reactions during the administration of *Bifidobaeterium* tetravaccine tablets (Live) and during the follow-up period.

## Discussion

In the present study, we found that RRTI children suffered from intestinal flora imbalance, which was manifested as a significant reduction in the number of *Bifidobacteria* and *Lactobacilli* and an increase in the number of *Escherichia coli*. At the same time, this study revealed that the number of *Bifidobacteria* and *Lactobacilli* in active stage of RRTI was lower than that in remission stage, while the number of *Escherichia coli* was significantly increased. Further, this suggested that the imbalance in intestinal flora is more prominent in the active stage than in the remission stage of RRTI, so we could speculate that the imbalance of intestinal flora is related to the episode of RRTI. After receiving *Bifidobaeterium* tetravaccine tablets (Live) treatment, the number of *Bifidobacteria* and *Lactobacilli* in children with RRTI was restored to the level of healthy controls, and was significantly higher compared to RRTI control group, suggesting that *Bifidobaeterium* tetravaccine tablets (Live) can effectively increase the number of *Bifidobacteria* and *Lactobacilli* in children with RRTI, and thereby to maintain the balance of intestinal micro-ecology.

Probiotics have been shown to have various immunomodulatory effects in the host [13–15]. Existing studies have revealed that early innate immunity of lung tissues against foreign infections is derived from the systemic regulation of intestinal flora via NOD-like receptors (NLRs), while the intestinal probiotics can also regulate IgA production by regulating pulmonary dendritic cells [16]. Intestinal probiotics enhance the host’s resistance to Pneumococcal pneumonia and *S. aureus* pneumonia [17, 18], as well as promote the antiviral effect in the lungs. Animal experiments have demonstrated that mice lacking probiotics have significant deficiencies in clearance of *K. pneumoniae* from the lungs [19]. In addition, intestinal probiotics can enhance the body’s antiviral immune response by stimulating inflammasome and inducing innate immune molecules [20], and they are involved in the regulation of pulmonary TH17-mediated antifungal immunity [21].

In this study, the follow-up demonstrated that the frequency of respiratory tract infections, duration of cough, duration of fever, duration and frequency of antibiotics use were significantly decreased in the RRTI intervention group compared with the control group. So far, numerous studies suggested that probiotic consumption can decrease the incidence of respiratory tract infections in children [22–25]. A latest systematic review including 23 RCTs and 6269 cases has shown that the frequency of respiratory tract infections was significantly reduced, the duration of respiratory tract infections was shortened in the probiotic intervention group [11]. So far, few studies have investigated changes of the intestinal flora in RRTI children. Peng et al. [26] have investigated the intestinal flora in 30 children with recurrent pneumonia and have found that the number of *Bifidobacteria* decreases and the number of *Escherichia coli* increases in children with recurrent pneumonia compared with healthy children, thus stimulating the imbalance of intestinal flora in children with recurrent pneumonia. In theory, Peng’s data suggested that probiotics should be given to promote intestinal flora balance during pneumonia treatment. Unfortunately, no other relevant studies have been conducted.

At present, there have been no reports on serious toxicities and side effects caused by probiotics due to its safety, and meanwhile infection and transmission resistance induced by probiotics have not been reported in China [27]. Similarly, in our study, no obvious adverse reactions were observed in all cases receiving oral *Bifidobaeterium* tetravaccine tablets (Live).

In summary, RRTI remains a major challenge for global public healthy, causing high morbidity and mortality among children [10]. Currently, probiotics have provided a new way for the prevention and treatment of children with RRTI. Nevertheless, since the curative effect of probiotics is limited to the influence of different strains, dose and treatment course, and the same strain may also have different effects on the children of different ages, its actual effectiveness should be verified using multi-center, large-scale and prospective clinical studies.
